# The mediating effects of perceived stress and exercise addiction risk on the relationship between body image state and emotional eating among college students

**DOI:** 10.3389/fpsyg.2026.1821570

**Published:** 2026-05-29

**Authors:** Yang Li, Qiang Guo, Lingli Jia

**Affiliations:** 1 School of Wushu, Chengdu Sport University, Chengdu, Sichuan, China; 2 School of Sports Training, Chengdu Sport University, Chengdu, Sichuan, China; 3 Academic Affairs Office, Chengdu Sport University, Chengdu, Sichuan, China

**Keywords:** body image state, college students, emotional eating, exercise addiction risk, perceived stress

## Abstract

**Objective:**

The association between body image state and eating behaviors among college students has garnered significant research attention. However, the processes through which body image state is associated with emotional eating via emotional and behavioral pathways remain unclear. This study examines the association between body image state and emotional eating among college students, as well as the serial mediating roles of perceived stress and exercise addiction risk in this association.

**Methods:**

A cross-sectional design was employed, with 1,307 college students (age 20.00 ± 1.18 years, 52.03% female) recruited from 15 provinces in China using stratified cluster sampling. Participants completed the Body Image State Scale, Perceived Stress Scale, Exercise Addiction Inventory, and Emotional Eating Scale. The Exercise Addiction Inventory assesses risk for exercise addiction rather than clinical diagnosis. Serial mediation effects were tested using the SPSS PROCESS macro, with 95% confidence intervals for indirect effects estimated via bootstrapping (5,000 resamples).

**Results:**

Body image state showed a significant negative correlation with emotional eating (*β* = −0.194, *p* < 0.001). Perceived stress (*β* = −0.036, 95% CI [−0.055, −0.017]) and exercise addiction risk (*β* = −0.042, 95% CI [−0.064, −0.022]) had significant indirect effects in the association between body image state and emotional eating. Furthermore, perceived stress and exercise addiction risk demonstrated a serial indirect effect in this association (*β* = −0.008, 95% CI [−0.013, −0.004]), accounting for 4.124% of the total effect.

**Conclusion:**

Body image state is associated with emotional eating among college students with perceived stress and exercise addiction risk showing a serial mediating indirect effect. This finding is consistent with emotional and behavioral pathways linking body image to eating behaviors, providing a theoretical reference for mental health interventions in this population.

## 1 Introduction

The university years represent a developmental period of heightened vulnerability to body image disturbances, emotional distress, and maladaptive eating behaviors, making college students a particularly relevant population for examining how these factors interrelate (Gilar-Corbi et al., 2025; Canonigo et al., 2024). Body image state refers to immediate perceptions and evaluations of one’s physical appearance at a given moment (Cash et al., 2002) and is closely associated with daily behaviors and psychological well-being (Zhou et al., 2024). Among its behavioral correlates, emotional eating, defined as eating to cope with negative emotions rather than physiological hunger (Houminer Klepar et al., 2024), has drawn sustained attention due to its links to both psychological and physical health.

However, the processes through which body image state is associated with emotional eating remain under-explored. Two potential pathways may account for this association. First, perceived stress may serve as an emotional pathway, given that body image concerns are associated with increased daily stress among college students (Anosike et al., 2025). Second, exercise addiction risk, a pattern of compulsive and inflexible exercise that reflects addictive tendencies rather than a clinical diagnosis, may function as a behavioral pathway (Weinstein and Szabo, 2023). Critically, whether these emotional and behavioral pathways operate sequentially to link body image state to emotional eating has not been examined.

Drawing on the transactional stress and coping framework (Lazarus and Folkman, 1984), this study proposes a unified model in which poor body image state functions as a cognitive stressor that is appraised as threatening and is associated with elevated perceived stress. In turn, perceived stress may motivate rigid, appearance-driven exercise as a coping behavior, potentially increasing exercise addiction risk. The subsequent depletion of self-regulatory resources associated with exercise addiction risk may then be associated with increased reliance on emotional eating as a maladaptive emotion regulation strategy. This sequential pathway, moving from cognitive appraisal to emotional response, to behavioral coping, and ultimately to eating behavior, integrates previously separate theoretical accounts into a coherent chain. A detailed theoretical rationale for each link is provided in the Literature Review.

This study aims to test this serial mediation model in a large sample of Chinese college students. Findings are expected to inform targeted interventions that address the interconnected cognitive, emotional, and behavioral mechanisms underlying emotional eating in this vulnerable population.

## 2 Literature review and hypothesis development

This study is guided by the transactional stress and coping framework (Lazarus and Folkman, 1984). Within this framework, the following sections develop the hypothesized sequential pathway from body image state (cognitive stressor) to perceived stress (emotional response) to exercise addiction risk (maladaptive coping behavior) and ultimately to emotional eating (coping outcome), drawing on complementary theoretical perspectives to elaborate specific mechanisms within this overarching model.

### 2.1 Body image state and emotional eating

The relationship between body image state and emotional eating serves as a crucial entry point for understanding abnormal eating behaviors among college students. Body image state refers to an individual’s momentary perception and evaluation of their physical appearance at a specific time, reflecting the dynamic and context-sensitive nature of bodily experience (Cash et al., 2002). Emotional eating denotes the tendency to consume food to cope with negative emotions rather than physiological hunger, representing an emotion-regulation-oriented coping behavior (Arnow et al., 1995). Exploring the relationship between these two constructs helps reveal how body image is associated with eating behaviors through emotional pathways.

Existing research provides empirical support for the association between body image state and emotional eating. A study of Chinese female college students found a significant negative correlation between poorer body image state and eating disorder symptoms, with negative emotions serving as parallel mediators in this relationship (Hou, 2025). While this study focused on clinical-level eating pathology, its findings suggest that body image states are associated with eating-related outcomes through emotional pathways, a logic applicable to emotional eating as a specific maladaptive eating pattern. Research directly examining emotional eating has similarly found associations with body image concerns. For instance, higher levels of body dissatisfaction were associated with more pronounced tendencies toward emotional eating among Chinese college students (Yi et al., 2025a). Another review study highlights a clear bidirectional association between emotional eating and body image disturbance, noting that individuals who engage in emotional eating may experience intensified dissatisfaction with their bodies (Fyodorova et al., 2021).

Theoretically, the process by which body image state is associated with emotional eating can be explained through stress coping theory. Lazarus and Folkman’s stress coping theory posits that individuals perceive stress when they evaluate external events as threatening and exceeding their coping resources (Lazarus and Folkman, 1984). Poor body image state can be viewed as a persistent threatening self-evaluation that is associated with increased stress perception (Brown et al., 2023). When stress arises, individuals require coping strategies to regulate emotions. Emotional eating serves as an emotion-regulation-oriented coping strategy, where individuals alleviate or escape negative emotional experiences through food consumption (Shireen et al., 2022). This theoretical logic is supported by research showing a strong association between body image distress and difficulties in emotion regulation (Monell, 2021). When individuals lack adaptive emotion regulation strategies, they may turn to specific maladaptive behaviors—such as emotional eating—to cope with distress. Consistently, a review study indicates that emotion regulation capacity plays a central role in the association between body image and eating behaviors (Abdoli et al., 2025). Thus, emotional eating can be understood as one specific, maladaptive manifestation of broader emotion regulation difficulties in the context of body image concerns.

However, existing research still has certain limitations. First, most studies focus on body image traits, that is, an individual’s consistent attitude toward their body, rather than body image states, that is, immediate experiences at a specific moment. Body image states exhibit situational variability and may better capture dynamic changes in daily life than traits (Scarpa, 2021), yet this perspective remains under-explored in the literature. Second, existing research predominantly examines direct relationships between body image and eating behaviors or investigates the role of single mediating variables, with limited attention to sequential transmission effects among multiple mediators. For instance, the complete psychological process, specifically how poor body image states are associated with subsequent behavioral choices through perceived stress and thereby relate to emotional eating, has not been systematically examined. Third, existing research has insufficiently addressed exercise-related variables. As a common behavior for improving physical appearance, exercise may play a significant role in this relationship, yet its underlying processes remain unclear.

The present study focuses on body image state rather than body image traits for several reasons. First, as a momentary, context-sensitive construct, body image state is conceptually more proximal to emotional eating, which often occurs as an impulsive response to immediate affective cues rather than as a planned behavior driven by stable attitudes. Second, the state-level approach captures dynamic fluctuations in appearance perceptions, complementing the predominantly trait-focused literature. Third, a state-level assessment aligns with the proposed sequential model, in which momentary body dissatisfaction is associated with perceived stress and subsequent behavioral responses over relatively short timeframes.

It is important to clarify why emotional eating, as distinct from clinically diagnosed eating disorders, represents a meaningful concern in non-clinical college populations. Emotional eating is associated with a range of adverse outcomes, including excess weight gain, elevated psychological distress, and poorer overall well-being (Houminer Klepar et al., 2024). Moreover, it may serve as a gateway to more severe eating pathology for some individuals, as habitual reliance on eating to regulate emotions can escalate into binge eating and other disordered eating patterns over time (Yi et al., 2025a). In college students specifically, emotional eating is prevalent and often coexists with academic stress and body image concerns, making it a relevant target for early identification and intervention. Thus, understanding the mechanisms through which body image state is associated with emotional eating carries both theoretical and practical significance, even in the absence of clinical pathology.

In summary, a stable association exists between body image state and emotional eating, which can be explained by stress coping theory. However, existing research on the mediating processes between these two factors remains fragmented, lacking systematic examination of the interplay among mediating variables. Based on this, the following hypothesis is proposed:

*H1*: Body image state is negatively associated with emotional eating among college students.

### 2.2 Body image state, perceived stress, and emotional eating

The association between body image state and emotional eating is not solely mediated through direct pathways; perceived stress may play a crucial mediating role in this relationship. Perceived stress refers to an individual’s subjective experience and evaluation of various stressful life events (Cohen et al., 1983), reflecting their cognitive judgment of the balance between personal coping resources and external demands. Exploring how body image state relates to emotional eating through perceived stress helps reveal the psychological processes by which body image distress is associated with emotion-driven eating behaviors.

The association between body image state and perceived stress can be explained by stress perception theory. Stress coping theory posits that perceived stress arises from an individual’s cognitive evaluation of events (Lazarus and Folkman, 1984). When an individual assesses concerns about physical appearance as a threat to self-worth and perceives insufficient coping resources to address this threat, perceived stress emerges (Ginis et al., 2012). Poor body image implies negative evaluations of one’s appearance, which may become a persistent source of self-threat, associated with activation of the stress perception system (Ginis et al., 2012). A study of Chinese female college students found a significant association between body image state and negative emotions (including perceived stress), with negative emotions mediating the relationship between body image and maladaptive eating outcomes (Hou, 2025). This finding provides empirical support for the association between body image state and perceived stress.

The relationship between perceived stress and emotional eating also rests on a solid theoretical foundation. According to emotion regulation theory, when individuals experience negative emotions and stress, they often employ specific coping strategies to alleviate these unpleasant feelings (Gross, 2002). As a convenient and effective form of emotional regulation, eating provides short-term comfort and pleasure, making it a common coping choice for stress (Alexatou et al., 2025). Existing research provides ample empirical evidence for this theoretical logic. A recent study of Saudi Arabian university students found a significant positive correlation between perceived stress and emotional eating, indicating that individuals with higher stress levels are more likely to cope with negative emotions through eating (El-Zayat et al., 2025). Another study similarly found that stress is associated with emotional eating, with the relationship between stress and emotional eating varying across sociocultural contexts, manifested through gender differences (Wang et al., 2022). These findings suggest that, while the general association between perceived stress and emotional eating is robust across populations, its specific manifestations may vary across sociocultural contexts. The implications of this cultural specificity for the present Chinese college student sample are further considered in the Discussion.

Further research has revealed more complex relational patterns among perceived stress, emotional eating, and body image. A cross-sectional study among Turkish adults found that emotional eating mediated the relationship between perceived stress and body appreciation, with perceived stress being associated with increased emotional eating behaviors, which in turn were associated with lower positive body evaluations (Ersoy et al., 2024). Although this study focused on body appreciation, the positive dimension of body image, rather than body image state, the revealed pathway, specifically stress associated with emotional eating associated with body evaluation, provides crucial theoretical reference for the present research. Considering the variable direction in this study, it can be inferred that poor body image state may be associated with an increased tendency toward emotional eating through elevated perceived stress.

From a risk factor perspective, difficulties in emotion regulation have been identified as a key factor linking body image concerns to maladaptive eating patterns (Morin and Meilleur, 2024). When individuals experience negative emotions stemming from body image dissatisfaction without effective coping strategies, they may rely on specific behaviors such as overeating to manage distress (Yi et al., 2025b). This perspective is consistent with the proposed mediating role of perceived stress: poor body image state is associated with elevated perceived stress, and individuals who lack adaptive emotion regulation strategies may turn to emotional eating as one specific yet maladaptive means of coping.

Synthesizing the above theoretical and empirical evidence, the following theoretical logic emerges: poor body image state, as a form of negative self-evaluation, is associated with activation of an individual’s stress perception system and elevated perceived stress levels. As a negative emotional experience, perceived stress is then associated with individuals adopting eating-based emotion regulation strategies, which in turn are associated with an increased tendency toward emotional eating. This pathway reveals the underlying process through which body image state is associated with emotional eating via perceived stress. Based on this, the following hypothesis is proposed:

*H2*: Perceived stress mediates the relationship between body image state and emotional eating among college students.

### 2.3 Body image state, exercise addiction risk, and emotional eating

Poor body image state is associated with emotional eating not only through the affective pathway of perceived stress but also via the behavioral pathway of exercise addiction risk. Exercise addiction refers to a compulsive, uncontrollable pattern of behavior toward regular exercise, characterized by the loss of flexibility in exercise habits. It becomes an obligation that individuals feel compelled to fulfill, often persisting despite physical injury or other negative life consequences. The present study assesses exercise addiction risk, reflecting individuals’ susceptibility to developing such patterns, rather than clinical diagnosis (Weinstein and Szabo, 2023). Exploring how body image state is associated with emotional eating through exercise addiction risk may reveal an additional process by which body image distress relates to maladaptive eating behaviors.

A key theoretical distinction in exercise addiction risk research aids in understanding how body image state relates to this behavioral pattern. Researchers categorize exercise addiction into primary and secondary types (Weinstein and Szabo, 2023). Primary exercise addiction centers on exercise itself as the core object of addiction; secondary exercise addiction serves other purposes, often linked to eating disorders or body image concerns, where exercise functions as a tool to achieve non-exercise goals (e.g., weight control, appearance enhancement) (Berczik et al., 2012). This distinction suggests that when individuals with poor body image perceive exercise as essential for appearance improvement, a once-healthy activity may be associated with compulsive instrumental behavior patterns, potentially increasing risk for secondary exercise addiction. In other words, body image concerns may act as a factor associated with elevated exercise addiction risk. Existing research supports this inference. A study among college students found that inflexible body image was significantly and positively associated with exercise addiction risk, indicating that rigid attitudes and negative evaluations of body image are correlates of exercise addiction (Zou et al., 2022). Another study similarly noted that excessive preoccupation with body image is significantly associated with exercise addiction risk. When fitness is viewed as a means to achieve specific aesthetic ideals rather than a way to improve health, the risk of exercise addiction is significantly higher (Bonfanti et al., 2022).

The association between exercise addiction risk and emotional eating can be understood from multiple perspectives. First, from a behavioral addiction viewpoint, exercise addiction risk and eating disorders may share psychological characteristics such as impulsivity, compulsivity, and difficulties in emotional regulation, making them prone to co-occurrence within the same population (Meyer et al., 2011). Second, from a self-regulation perspective, the compulsive and over-controlling behaviors associated with exercise addiction risk may continuously deplete an individual’s limited self-regulatory resources (Baumeister et al., 2018). Once these resources are exhausted, individuals find it more difficult to regulate emotions and suppress eating urges when facing stress or negative emotions, a pattern associated with emotional eating (Muraven and Baumeister, 2000). Empirical research supports these theoretical inferences. A cross-sectional study among adolescents revealed a significant positive correlation between exercise addiction risk and eating disorder symptoms, with this association mediated through multiple pathways including psychological distress, insomnia, and body image concerns (Ahorsu et al., 2023). Although this study examined clinical-level eating pathology, the shared psychological features between exercise addiction risk and maladaptive eating (e.g., impulsivity, compulsivity, emotion regulation difficulties) are also relevant to subclinical emotional eating patterns. An alternative explanation is that greater exercise addiction risk could be associated with higher exercise volume, which may increase physiological hunger and overall food consumption rather than emotionally driven eating. However, the Emotional Eating Scale specifically assesses eating in response to emotional states rather than hunger, and exercise addiction risk reflects compulsive behavioral patterns rather than simply high exercise volume.

Synthesizing the above analysis, theoretically, poor body image, as a form of negative self-evaluation, may be associated with individuals viewing exercise as a tool for improving appearance. When this instrumental motivation becomes excessively tied to body image concerns, exercise behavior may be associated with secondary exercise addiction patterns. The psychological resource depletion and physiological imbalance accompanying exercise addiction risk may be associated with weakened control over eating urges, which in turn is associated with increased emotional eating tendencies. This pathway reveals the underlying process through which body image state is associated with emotional eating via exercise addiction risk. Based on this, the following hypothesis is proposed:

*H3*: Exercise addiction risk mediates the relationship between body image state and emotional eating among college students.

### 2.4 Body image state, perceived stress, exercise addiction risk, and emotional eating

The preceding sections have separately examined the potential mediating roles of perceived stress and exercise addiction risk in the relationship between body image state and emotional eating. However, examining these two variables in isolation may overlook the interplay between emotional and behavioral pathways that is likely to occur in real-world settings. Emotional eating represents a prevalent yet maladaptive coping pattern in college populations, associated with weight gain, elevated psychological distress, and reduced well-being, even in the absence of clinically diagnosed eating disorders. From an intervention perspective, understanding whether perceived stress and exercise addiction risk operate independently or sequentially is critical: if a sequential pathway exists, interventions targeting only stress or only exercise behavior may be insufficient to disrupt the link between body image concerns and emotional eating. Elucidating these interconnected mechanisms can help identify multiple, potentially synergistic points of intervention, such as stress management training combined with the promotion of flexible, intrinsically motivated exercise, to reduce reliance on eating as an emotion regulation strategy. It should be noted that the proposed sequence is grounded in theoretical reasoning (e.g., stress coping theory suggests that emotional appraisal precedes coping behaviors) rather than empirical temporal evidence; the cross-sectional design of the current study cannot confirm the causal order of these variables.

From a temporal perspective, stress perception, as an emotional experience variable, is theorized to precede exercise addiction risk, a behavioral-level variable. According to stress coping theory, when individuals encounter threatening events, emotional stress perception arises first, followed by the initiation of coping behaviors (Lazarus and Folkman, 1984). Poor body image, as a self-threat, is associated with activation of the individual’s stress perception system and elevated stress levels (Ginis et al., 2012). The selection and execution of exercise behaviors, however, constitute a coping process that, according to this theoretical framework, is initiated after stress perception has been generated. Empirical research has documented a significant positive association between stress and exercise addiction risk in college students (Wang et al., 2025, Soraci et al., 2024), consistent with the theoretical expectation that these two constructs are linked, though the temporal order proposed here is derived from theory rather than longitudinal evidence. Based on this theoretical reasoning, perceived stress is hypothesized to precede exercise addiction risk in the serial mediation model.

Theoretically, the association between stress perception and exercise addiction risk can be further elucidated through Self-Determination Theory. This theory distinguishes between autonomous motivation and controlled motivation. Autonomous motivation stems from an individual’s intrinsic interests and values, while controlled motivation arises from external pressures or internal pressures, such as shame or anxiety (Ryan and Deci, 2000). The stress associated with body image issues typically does not become interest in the health behavior itself but instead may be associated with compulsive, controlled motivation driven by shame and anxiety (Sabiston et al., 2014). Under this control-based motivation, exercise behavior may lose its intrinsic enjoyment and be associated with rigid, compulsive instrumental actions that may relate to exercise addiction risk (Dinardi et al., 2021). In other words, perceived stress may provide psychological conditions associated with exercise addiction risk through shaping control-based motivation. Existing research provides indirect support for this logic. A study of college students found a significant positive correlation between stress and exercise addiction risk, suggesting that individuals with higher stress levels may be more likely to report excessive dependence on exercise (Wang et al., 2025).

The interactive effect between perceived stress and exercise addiction risk further strengthens the theoretical rationale for both serving as sequential mediators. When exercise addiction risk is present, it may be associated with increased tendencies toward emotional eating. From a self-depletion perspective, exercise addiction risk manifests as excessive control and rigid persistence in exercise behavior. This high-intensity self-control may consume substantial psychological resources (Baumeister and Vonasch, 2015). When individuals experience resource depletion associated with exercise addiction risk, they may find it harder to control eating impulses when facing negative emotions, a pattern associated with emotional eating (Graham and Bray, 2015). Furthermore, exercise addiction risk itself may become associated with new stressors. The craving for exercise opportunities, anxiety when unable to exercise, and physical injuries from exercise may all be associated with increased perceived stress, potentially creating a cycle (Martín-Rodríguez et al., 2024). This mutual reinforcement between perceived stress and exercise addiction risk suggests a sequential pathway in their associations with emotional eating.

**Figure 1:**
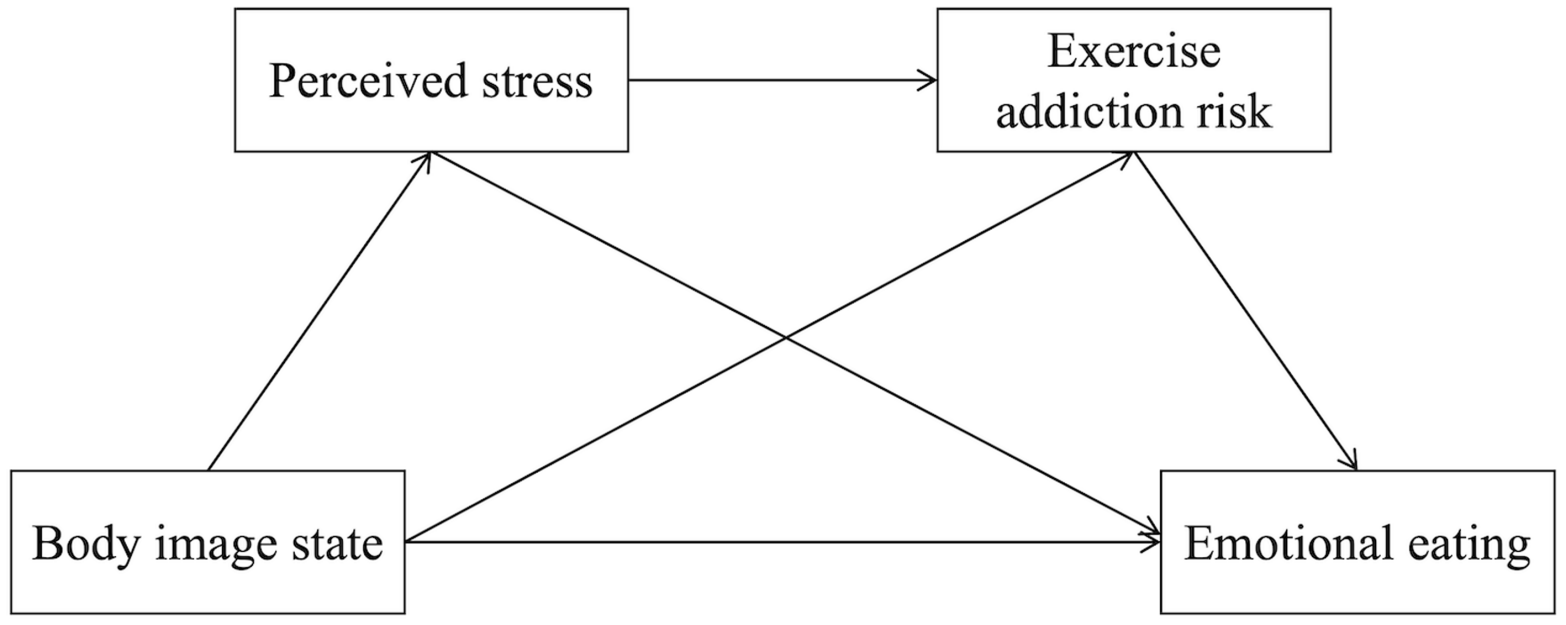
Conceptual model of the serial mediation effect.

In summary, perceived stress and exercise addiction risk may form a serial mediating pathway between body image state and emotional eating. Specifically, poor body image state is associated with elevated perceived stress levels. As a negative emotional experience, perceived stress may be associated with individuals engaging in exercise aimed at improving appearance. Under conditions associated with control-seeking motives, this may relate to exercise addiction risk. The psychological resource depletion and physiological imbalance accompanying exercise addiction risk may be associated with weakened control over eating impulses, which in turn is associated with increased emotional eating tendencies. This sequential pathway illustrates a psychological process extending from cognitive evaluation (body image) to emotional experience (stress perception), then to behavioral patterns (exercise addiction risk), and finally to associations with eating behavior (emotional eating). Based on this, the following hypothesis is proposed:

*H4*: Perceived stress and exercise addiction risk mediate the relationship between body image state and emotional eating in a chain-like manner. The conceptual model is illustrated in Figure 1.

## 3 Methodology

### 3.1 Procedures and participants

This study employed a cross-sectional design. Between November 1 and November 30, 2025, a stratified sampling method was used to recruit 1,400 adult college students from 15 provinces across five regions of China: eastern, central, western, southern, and northern. Stratification was performed by geographical region (five strata corresponding to the five regions) and by university type (comprehensive, normal, and technical/vocational universities) within each region. This dual stratification aimed to capture diversity in economic development, educational resources, and cultural norms that may influence body image and eating behaviors. Within each stratum, cluster units were defined as intact classes (natural class groups) from randomly selected universities. Specifically, in each of the 15 provinces, we first randomly selected one university from each university type present in that province. From each selected university, we then randomly sampled two to three classes (cluster units) from the first- to fourth-year student population, ensuring proportional representation across grades. All students in the selected classes were invited to participate, yielding a target sample of approximately 100 students per province. Class advisors were responsible for distributing the questionnaires. Prior to distribution, the research team provided training to teachers on key aspects of questionnaire administration, including voluntary participation, the right to withdraw, and confidentiality requirements.

Following the training, class advisors thoroughly explained the questionnaire’s structure to students, emphasizing the principle of voluntary participation and the right to withdraw. The questionnaire was then distributed to students for completion via an online platform.^1^ This electronic survey comprised five sections: basic information (grade level, gender, height, weight), the Body Image State Scale, the Perceived Stress Scale, the Exercise Addiction Risk Scale, and the Emotional Eating Scale. To ensure data validity, attention check items (e.g., “If this question is incorrect, select number 1”) were inserted in both the first and second halves of the questionnaire. Certain positively worded items were reverse-scored to facilitate identification of invalid responses. Prior to data collection, all participants signed an electronic informed consent form. After data collection, all information was anonymized to ensure confidentiality. The anonymized dataset will be securely stored in accordance with institutional ethics guidelines and data management policies, remaining accessible for research purposes for at least five years post-publication. Qualified researchers may request access to the data upon signing a formal data-sharing agreement, which ensures participant privacy protection and adherence to original informed consent terms.

Following data collection, 93 invalid responses were excluded based on predefined criteria: (1) abnormally short or long completion times; (2) responses failing attention check items. The final sample comprised 1,307 participants, yielding a valid response rate of 93.36%. Participants ranged in age from 18 to 24 years (M = 20.00, SD = 1.18). The sample included 627 males (47.97%) and 680 females (52.03%). Grade distribution was: 435 first year (33.28%), 456 s year (34.89%), 308 third year (23.57%), and 108 fourth year students (8.26%). Regarding household registration, 598 participants (45.75%) were from urban areas and 709 (54.25%) from rural areas.

### 3.2 Ethics approval and consent to participate

This study was conducted in strict compliance with the ethical principles of the Declaration of Helsinki. The research protocol was approved by the Ethics Committee of Chengdu Sport University (Approval No.: CTYLL2025202). All participants signed an electronic informed consent form via an online platform prior to formal participation. The consent form detailed the study objectives, the principle of voluntary participation, confidentiality agreements, and the right to withdraw from the study at any time without penalty. To ensure participant privacy and security, data were anonymized during both collection and analysis. The final dataset contained no personally identifiable information.

### 3.3 Measurement tools

#### 3.3.1 Body Image State Scale

Body image state was assessed using the Body Image State Scale (Cash et al., 2002). This unidimensional scale consists of six items (e.g., “How satisfied are you with your appearance?”). Items are rated on a 9-point Likert scale ranging from 1 (Very dissatisfied) to 9 (Very satisfied), with higher total scores indicating more positive body image states. The Chinese version of the BISS has been shown to have good internal consistency and structural validity among Chinese people (An et al., 2026). In this study, the scale demonstrated good internal consistency with Cronbach’s *α* = 0.892.

#### 3.3.2 Perceived Stress Scale

Perceived stress was assessed using the Perceived Stress Scale (Cohen et al., 1983), which measures the degree to which situations in one’s life are appraised as stressful over the past month. This unidimensional scale comprises 14 items (e.g., “How often in the past month have you felt overwhelmed?”). Items are rated on a 5-point Likert scale, with higher total scores indicating greater perceived stress. The simplified Chinese version of the PSS-10 (PSS-C-10) has demonstrated robust reliability and a stable two-factor structure in Chinese university students (Lu et al., 2017). In this study, the scale demonstrated excellent internal consistency with Cronbach’s *α* = 0.953.

#### 3.3.3 Exercise Addiction Inventory

Exercise addiction risk was assessed using the Exercise Addiction Inventory (EAI) (Griffiths et al., 2005). This 6-item scale assesses six core components of addiction: salience, conflict, emotional regulation, tolerance, withdrawal symptoms, and relapse. Items are rated on a 5-point Likert scale ranging from 1 (Strongly disagree) to 5 (Strongly agree). The EAI measures addictive symptoms and tendencies in exercise behavior, reflecting the level of exercise addiction risk rather than providing a clinical diagnosis. The EAI does not provide a clinical diagnosis of exercise addiction; rather, it reflects the severity of addictive symptoms in the context of exercise behavior. In non-clinical samples such as ours, higher total scores are interpreted as indicating a higher risk profile or a stronger tendency toward addictive exercise patterns (hereafter referred to as “exercise addiction risk”). Higher total scores indicate greater exercise addiction risk. The Chinese version of the EAI has been validated among Chinese college students, showing adequate reliability and validity (Wang et al., 2024). In this study, the scale demonstrated good internal consistency with Cronbach’s *α* = 0.898.

#### 3.3.4 Emotional eating scale

Emotional eating was assessed using the emotional eating subscale of the Dutch Eating Behavior Questionnaire (Van Strien et al., 1986). This 13-item scale measures the tendency to eat in response to emotional states such as anger, boredom, disappointment, and tension (e.g., “Do you have the desire to eat when you are irritated?”). Items are rated on a 5-point Likert scale ranging from 1 (Never) to 5 (Always), with higher scores indicating more frequent emotional eating. The Chinese version of the DEBQ has been validated among Chinese undergraduates, demonstrating good factorial validity and internal consistency (Zhu et al., 2013). In this study, the scale demonstrated excellent internal consistency with Cronbach’s α = 0.932.

### 3.4 Data analysis

The data analysis procedure comprised three steps:

1. Common method bias assessment: Harman’s one-factor test was employed to evaluate potential common method bias.
2. Descriptive statistics and correlation analysis: Means, standard deviations, and Pearson correlation coefficients were calculated for all study variables (body image state, perceived stress, exercise addiction risk, and emotional eating).
3. Chain mediation analysis: A chain mediation model was constructed using the PROCESS macro (version 4.0) for SPSS 25.0 (Model 6). This model examined the mediating effects of perceived stress and exercise addiction risk in the association between body image state and emotional eating. Gender, grade level, and body mass index were included as covariates. Indirect effects were tested using bootstrap resampling with 5,000 iterations, generating 95% confidence intervals for the indirect effects.
4. Selection of covariates: Gender, grade level, and body mass index (BMI) were included as covariates based on prior research showing their associations with body image, perceived stress, exercise behavior, and emotional eating among college students (Wang et al., 2022). No other variables were available for adjustment. We acknowledge that additional unmeasured confounders may exist; this limitation is addressed in the Discussion (Section 5.7).

## 4 Results

### 4.1 Common method bias test

Because the data were collected via self-report questionnaires, Harman’s single-factor test was conducted to assess potential common method bias. An exploratory factor analysis was performed on all items from the Body Image State Scale, Perceived Stress Scale, Exercise Addiction Inventory, and Emotional Eating Scale. Unrotated principal component analysis revealed that the first factor accounted for 28.786% of the total variance, which is below the recommended threshold of 40%. These results suggest that common method bias is not a severe concern in this study; however, as discussed in Section 5.7, *post hoc* assessments such as the Harman test do not definitively rule out some risk of inflated associations.

### 4.2 Descriptive statistics and correlations

Descriptive statistics and Pearson correlation analyses were conducted for all study variables (body image state, perceived stress, exercise addiction risk, and emotional eating). The results are presented in Table 1. All variables showed significant intercorrelations. Body image state was negatively correlated with emotional eating (*r* = −0.194, *p* < 0.01), perceived stress (*r* = −0.317, *p* < 0.01), and exercise addiction risk (*r* = −0.413, *p* < 0.01). Perceived stress was positively correlated with exercise addiction risk (*r* = 0.312, *p* < 0.01) and emotional eating (*r* = 0.184, *p* < 0.01). Exercise addiction risk was positively correlated with emotional eating (*r* = 0.201, *p* < 0.01).

**Table 1:** Descriptive statistics and correlation analysis of variables.

Variable	Mean	Standard deviation	Body image state	Perceived stress	Exercise addiction risk	Emotional eating
Body image state	29.396	8.954	1			
Perceived stress	31.661	10.240	−0.317**	1		
Exercise addiction risk	19.565	4.826	−0.413**	0.312**	1	
Emotional eating	42.562	9.186	−0.194**	0.184**	0.201**	1

**p < 0.01.

### 4.3 Mediating effect analysis

To examine the associations between body image state, perceived stress, exercise addiction risk, and emotional eating, a series of regression analyses were conducted. Body image state, perceived stress, and exercise addiction risk were entered as independent variables, with emotional eating as the dependent variable (Table 2).

**Table 2:** Regression analysis of body image state, perceived stress, and exercise addiction risk on emotional eating.

Variant	Emotional eating			
	β	T	F	R^2^adj
Body image state	−0.199	−7.140***	50.977	0.037
Perceived stress	0.184	6.773***	45.878	0.033
Exercise addiction risk	0.201	7.413***	54.955	0.040

R^2^adj is the adjusted R-squared value. ***p < 0.001.

Results indicated that body image state was negatively associated with emotional eating (*β* = −0.199, t = −7.140, *p* < 0.001), while perceived stress (*β* = 0.184, t = 6.773, *p* < 0.001) and exercise addiction risk (*β* = 0.201, *t* = 7.413, *p* < 0.001) were positively associated with emotional eating. After controlling for gender, grade level, and BMI, the chained mediation model remained significant, indicating the robustness of the findings.

The chained mediating effects of perceived stress and exercise addiction risk in the relationship between body image state and emotional eating were examined using the PROCESS macro (Version 4.0; Model 6) for SPSS 25.0, with gender and grade level included as covariates. Indirect effects were estimated using bootstrap resampling with 5,000 iterations, generating 95% bias-corrected confidence intervals (CIs).

As shown in Table 3, body image state was significantly associated with emotional eating (*β* = −0.194, 95% CI [−0.247, −0.141]), supporting Hypothesis 1. Perceived stress showed a mediating indirect effect in the association between body image state and emotional eating (indirect effect = −0.036, 95% CI [−0.055, −0.017]), supporting Hypothesis 2. Exercise addiction risk also showed a mediating indirect effect in this association (indirect effect = −0.042, 95% CI [−0.064, −0.022]), supporting Hypothesis 3. Furthermore, perceived stress and exercise addiction risk showed a serial indirect effect in the association between body image state and emotional eating (indirect effect = −0.008, 95% CI [−0.013, −0.004]), supporting Hypothesis 4.

**Table 3:** Intermediary effect values and effect sizes.

Effect pathway	Effect	BOOT SE	BOOT LLCI	BOOT ULCI	Relative mediation effect
Total effect	−0.194***	0.027	−0.247	−0.141	100%
Direct effect	−0.108***	0.030	−0.167	−0.049	55.670%
Total indirect effect	−0.086***	0.015	−0.116	−0.057	44.330%
Indirect effect 1 (perceived stress)	−0.036***	0.010	−0.055	−0.017	18.557%
Indirect effect 2 (→ exercise addiction risk)	−0.042***	0.011	−0.064	−0.022	21.649%
Indirect effect 3 (perceived stress and → exercise addiction risk)	−0.008***	0.002	−0.013	−0.004	4.124%

Indirect effect 1: Body image state → Perceived stress → Emotional eating; Indirect effect 2: Body image state → Exercise addiction risk→ Emotional eating; Indirect effect 3: Body image state → Perceived stress → Exercise addiction risk→ Emotional eating. ***p < 0.001.

**Figure 2:**
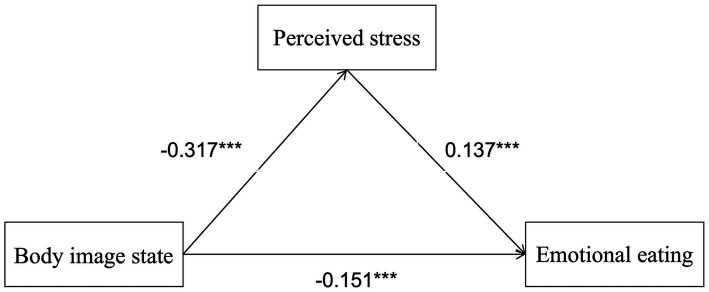
Model of mediating roles of perceived stress between body image state and emotional eating.

**Figure 3:**
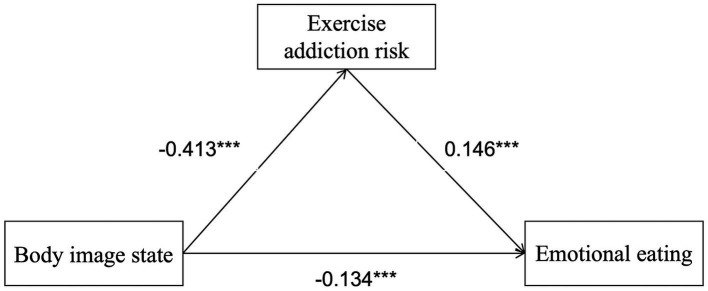
Model of mediating roles of exercise addiction risk between body image state and emotional eating.

**Figure 4:**
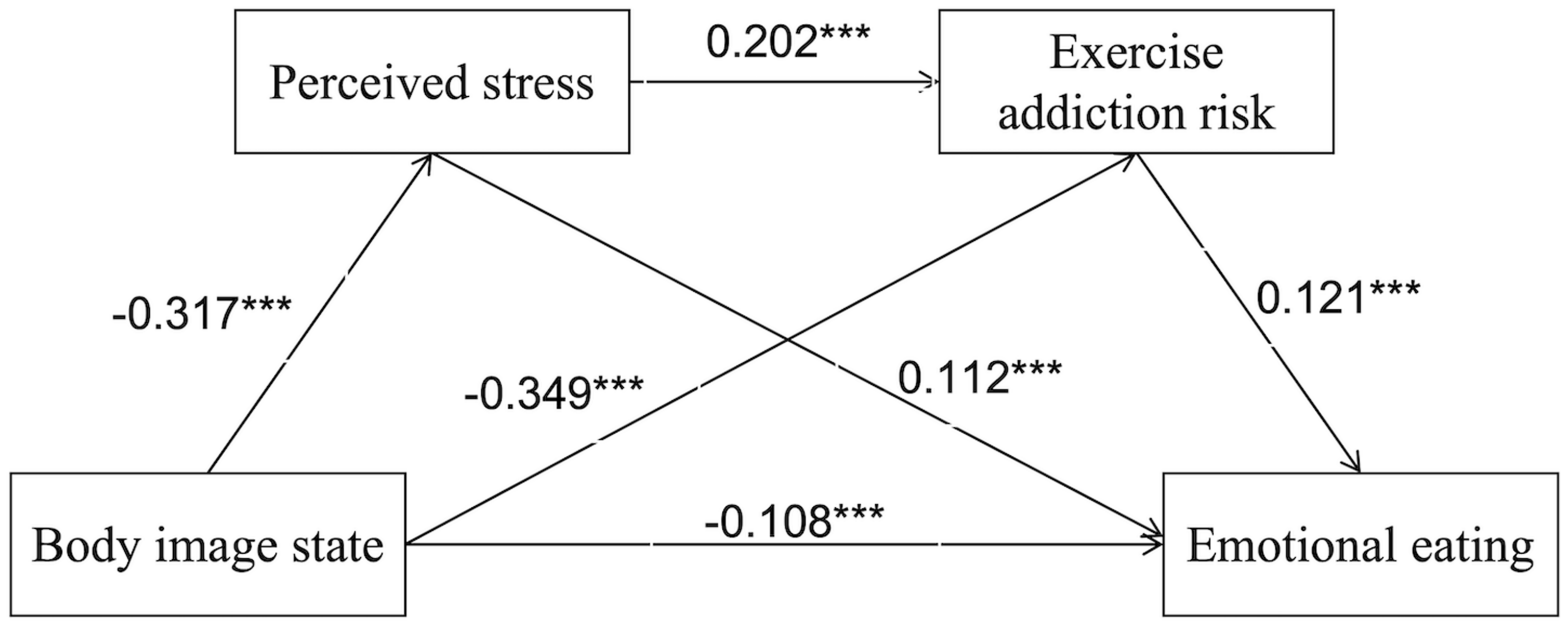
Model of chain mediating roles of perceived stress and exercise addiction risk between body image state and emotional eating.

All indirect effects were statistically significant, as indicated by their 95% confidence intervals excluding zero. The total effect of body image state on emotional eating was −0.194, which consisted of a direct effect of −0.108 (55.67% of the total effect) and a total indirect effect of −0.086 (44.33%). The specific indirect effects were as follows: through perceived stress alone (Path 1: body image state → perceived stress → emotional eating) = −0.036 (18.56% of the total indirect effect); through exercise addiction risk alone (Path 2: body image state → exercise addiction risk → emotional eating) = −0.042 (21.65% of the total indirect effect); and through perceived stress and exercise addiction risk in sequence (Path 3: body image state → perceived stress → exercise addiction risk → emotional eating) = −0.008 (4.12% of the total indirect effect). Path diagrams illustrating these effects are presented in Figures 2–4.

### 4.4 Alternative model testing

Because cross-sectional data cannot establish temporal order, we tested two additional mediation models to compare with our hypothesized serial model (Model 6).

Parallel mediation model (PROCESS Model 4). With perceived stress and exercise addiction risk as parallel mediators, both indirect effects were statistically significant: via perceived stress (effect = −0.0355, 95% CI [−0.0561, −0.0166]) and via exercise addiction risk (effect = −0.0501, 95% CI [−0.0767, −0.0248]).

Reversed serial mediation model (PROCESS Model 6). With the order: body image state → exercise addiction risk → perceived stress → emotional eating, the reversed serial indirect effect was also significant (effect = −0.0101, 95% CI [−0.0167, −0.0045]).

These results indicate that the data are statistically consistent with both our hypothesized serial order (body image state → perceived stress → exercise addiction risk → emotional eating) and the reversed order. Therefore, cross-sectional data cannot uniquely support one temporal sequence over another. The hypothesized indirect pathways reported in Table 3 remain statistically robust, but their interpretation as evidence of causal ordering is not warranted. Given that both the hypothesized and reversed serial indirect effects were statistically significant, the data are consistent with multiple temporal orders. Our model selection is guided by theory (stress coping theory suggests stress perception precedes coping behaviors), not by empirical discrimination.

## 5 Discussion

### 5.1 Key findings

This study examined the relationship between body image state and emotional eating among college students, exploring the sequential mediating roles of perceived stress and exercise addiction risk. Drawing on stress coping theory, the primary-secondary distinction in exercise addiction research, self-determination theory, and self-depletion theory, a sequential mediation model was constructed to test the pathway through which body image state is associated with emotional eating via perceived stress and exercise addiction risk. Employing a cross-sectional design, the study surveyed a college student population and tested four proposed hypotheses.

The results supported all hypotheses. First, body image state was negatively associated with emotional eating among college students, suggesting that individuals with poorer body image reported more frequent emotional eating behaviors. Second, perceived stress showed a mediating indirect effect in the association between body image state and emotional eating. Poorer body image state was associated with higher perceived stress levels, which in turn were associated with increased emotional eating. Third, exercise addiction risk showed a mediating indirect effect in the association between body image state and emotional eating. Poorer body image state was associated with greater exercise addiction risk, which was in turn associated with emotional eating. Fourth, and central to this study, perceived stress and exercise addiction risk showed a serial indirect effect, consistent with a sequential mediating pathway between body image state and emotional eating. By examining this sequential pathway, the present study extends prior research in two respects. First, it moves beyond single-mediator models to test whether emotional and behavioral pathways operate in sequence rather than independently. Second, it identifies exercise addiction risk as a behavioral mediator, a variable that has received limited attention in the body image–eating behavior literature despite its conceptual relevance.

The relatively small effect sizes observed in this study should be acknowledged. As a complex eating behavior, emotional eating is influenced by multiple factors, and body image state explains only a limited amount of variance on its own—a common finding in non-clinical samples. In non-clinical populations, associations between variables typically exhibit modest effect sizes, which does not diminish the theoretical significance of the findings but does require careful interpretation.

To illustrate the magnitude of these associations in a statistically meaningful way, the standardized coefficients indicate that a one-standard-deviation increase in positive body image state is associated with a 0.19-standard-deviation decrease in emotional eating scores in this sample. Although modest at the individual level, when considered across the entire population of college students, such an association could reflect a non-trivial pattern at the group level. The serial indirect effect, accounting for 4.12% of the total effect, is statistically robust (95% CI excludes zero) and provides evidence for a previously untested sequential pathway. Future research using longitudinal or experimental designs is needed to determine whether these associations reflect causal ordering and to identify subgroups in which these patterns may be stronger.

### 5.2 Direct effects of body image state on emotional eating

This study found that body image state was significantly and negatively associated with emotional eating among college students. That is, individuals with poorer body image states were more likely to report coping with negative emotions through eating. This result supports Hypothesis 1 and aligns with existing research findings.

Previous research provides empirical foundations for this finding. A study among college students indicated a significant negative correlation between body image dissatisfaction and disordered eating behaviors, with over half of participants expressing dissatisfaction with their body image and exhibiting higher levels of disordered eating (Jiménez-Limas et al., 2022). A cross-sectional survey of Chinese college students also found that higher levels of body image dissatisfaction were associated with more pronounced tendencies toward emotional eating (Yi et al., 2025a). Building upon these findings, the present study extends the focus from body image traits to body image states, emphasizing the direct association between immediate body image experiences and eating behaviors. This perspective helps capture the dynamic fluctuations of body image in daily life, addressing the limitation of trait measurements in reflecting situational variability.

Theoretically, this finding can be explained by stress coping theory. Lazarus and Folkman’s stress coping theory posits that when individuals assess external events as threatening and exceeding their coping resources, they perceive stress and subsequently employ coping strategies to regulate emotions (Lazarus and Folkman, 1984). Poor body image states can be viewed as a persistent threatening self-evaluation that is associated with increased stress perception. When individuals lack adaptive emotional regulation strategies, eating serves as a convenient and effective means of emotional regulation, providing short-term comfort and pleasure, and may thus be a common choice for coping with negative emotions. This explanation is supported by systematic review studies, which indicate a close association between body image distress and difficulties in emotional regulation, and that this association is closely related to disordered eating behaviors (Abdoli et al., 2025).

### 5.3 The mediating role of perceived stress

This study found that perceived stress showed a mediating indirect effect in the association between body image state and emotional eating among college students, supporting Hypothesis 2. This result is consistent with the view that poor body image state is associated with emotional eating not only directly but also indirectly through elevated perceived stress levels.

This finding aligns with existing research. A study of first-year college students reported similar results, showing a significant positive correlation between perceived stress and emotional eating (Musyafira and Dewi, 2024). Mendia found that negative emotions mediated the relationship between body image and eating disorder symptoms (Mendia et al., 2021), while Ersoy identified a pathway through which perceived stress is associated with body appreciation via emotional eating (Ersoy et al., 2024). Building upon this foundation, the present study explicitly positions perceived stress between body image state and emotional eating, is consistent with a transmission chain from cognitive evaluation to emotional experience to eating behavior.

Theoretically, this mediating pathway can be explained by stress coping theory. Lazarus and Folkman (1984) noted that individuals perceive stress when they evaluate events as threats to their self-worth. Poor body image, as a self-threat, is associated with activation of an individual’s stress perception system. When stress arises, individuals require coping strategies to regulate emotions. Eating, as a convenient emotional regulation method, becomes a common response associated with stress. Excessive focus on body image is associated with heightened negative emotions, and difficulties in emotion regulation may impair individuals’ healthy coping skills, potentially leading them to rely on emotional eating for temporary emotional relief (Harrison et al., 2016). This study’s finding of a mediating indirect effect of perceived stress is consistent with the broader literature on emotion regulation difficulties (Fahim, 2025), in which emotional eating is understood as one specific maladaptive emotion regulation strategy. The empirical data are consistent with a pathway through which body image distress is associated with increased emotional eating via elevated perceived stress levels.

### 5.4 The mediating role of exercise addiction risk

This study found that exercise addiction risk showed a mediating indirect effect in the association between body image state and emotional eating among college students, supporting Hypothesis 3. This result is consistent with the view that poor body image state is also associated with eating behaviors through behavioral pathways: negative evaluations of physical appearance may be associated with individuals engaging in exercise aimed at improving appearance. When exercise motivation becomes excessively tied to body image concerns, exercise may be associated with exercise addiction risk, which in turn is associated with emotional eating.

This finding corroborates existing research. Zou et al. found that inflexible body image was significantly and positively associated with exercise addiction risk (Zou et al., 2022). Another study also reported a significant positive correlation between exercise addiction risk and eating disorders, potentially mediated through multiple pathways including psychological distress (Arusoglu et al., 2025). The present study integrates body image state, exercise addiction risk, and emotional eating into a unified framework, identifying a pathway that is consistent with a sequence from cognitive evaluation to maladaptive exercise behavior to emotion-driven eating.

Theoretically, this mediating pathway can be understood from two perspectives. First, the primary-secondary distinction in exercise addiction research differentiates between primary and secondary exercise addiction, with the latter serving external goals such as appearance improvement (Weinstein and Szabo, 2023). Individuals with poor body image may perceive exercise as a tool for appearance enhancement, potentially increasing susceptibility to secondary exercise addiction risk (Abi Karam et al., 2025). A 2024 review synthesizing existing evidence confirmed a clear and stable association between body image and exercise addiction risk in adults (Guo et al., 2025). Second, self-depletion theory suggests that the excessive control associated with exercise addiction risk may deplete psychological resources, making it harder for individuals to regulate eating impulses when resources are depleted (Baumeister et al., 2018). Another study also indicates that exercise addiction risk and eating disorders may share behavioral addiction characteristics, making them prone to co-occurrence (Lev Arey et al., 2023).

This study’s finding of the mediating role of exercise addiction risk expands the research perspective on the relationship between body image and eating behaviors. It suggests that not all exercise behaviors carry positive significance; when exercise motivation becomes excessively tied to body concerns, healthy exercise may be associated with addictive patterns and may relate to eating behaviors.

### 5.5 Chain mediating effect of perceived stress and exercise addiction risk

The core finding of this study is the identification of a serial indirect effect consistent with a chain mediation model of perceived stress and exercise addiction risk in the association between body image state and emotional eating among college students, supporting Hypothesis 4. This result is consistent with a psychological and behavioral chain linking body image distress to emotional eating: poor body image state is associated with elevated perceived stress levels, which in turn are associated with exercise addiction risk, and ultimately associated with emotional eating. However, because cross-sectional data cannot establish temporal precedence, we tested a reversed serial order (body image state → exercise addiction risk → perceived stress → emotional eating). That reversed serial indirect effect was also statistically significant (Section 4.4). Consequently, the data do not allow us to conclude whether perceived stress precedes exercise addiction risk or vice versa. Our hypothesized sequence remains theoretically plausible—grounded in stress coping theory and self-determination theory—but its temporal directionality cannot be empirically confirmed with the present design. Future longitudinal or experimental studies are needed to test the causal ordering of these variables.

From a temporal perspective, stress perception, as an emotional experience variable, is theorized to precede exercise addiction risk, a behavioral-level variable. Stress coping theory indicates that when individuals encounter threatening events, emotional stress perception arises first, followed by the initiation of coping behaviors (Lazarus and Folkman, 1984). Poor body image, as a self-threat, is associated with activation of the individual’s stress perception system and elevated stress levels. The selection and execution of exercise behaviors, however, constitute a coping process that may occur after stress perception has been generated. Therefore, the temporal sequence of stress perception preceding exercise addiction risk is theoretically plausible.

From a theoretical perspective, the association between stress perception and exercise addiction risk can be elucidated through Self-Determination Theory. Deci and Ryan distinguish between autonomous and controlled motivation. When individuals perceive stress associated with poor body image, this stress may be related to controlled motivation (Ryan and Deci, 2000). Under conditions associated with such motivation, exercise behavior may lose its intrinsic pleasure and be associated with rigid, compulsive instrumental actions that may relate to exercise addiction risk. The finding that stress and exercise addiction risk show a significant positive correlation provides indirect support for this association (Soraci et al., 2024).

How exercise addiction risk subsequently relates to emotional eating can be explained by self-depletion theory. Baumeister noted that individuals possess limited self-control resources, and excessive control may deplete psychological resources (Baumeister et al., 2018). Exercise addiction risk manifests as rigid persistence and high control over exercise behavior, and this sustained self-control may consume substantial psychological resources. When individuals experience resource depletion associated with exercise addiction risk, they may find it more difficult to control eating impulses when facing negative emotions, a pattern associated with emotional eating.

This study found evidence consistent with a chain mediating pathway, integrating stress coping theory, self-determination theory, and self-depletion theory. It examined a theoretical model spanning from cognitive evaluation (body image) to emotional experience (stress perception) to behavioral patterns (exercise addiction risk), and ultimately to eating behavior (emotional eating). This finding addresses existing research gaps in examining the interlinked dynamics among mediating variables, identifying processes that are consistent with the view that body image is associated with emotional eating through sequential associations. Rather than operating independently, perceived stress and exercise addiction risk may form a sequential chain that relates to individuals’ eating behavior tendencies. This finding contributes to the literature by demonstrating that the emotional and behavioral mechanisms linking body image to eating behaviors are not merely parallel processes but may be interconnected in theoretically meaningful ways, with implications for how interventions are sequenced.

### 5.6 Theoretical contributions

This study makes several theoretical contributions by constructing and examining a serial mediation model linking perceived stress and exercise addiction risk with body image state and emotional eating.

First, this research extends the perspective of body image studies from traits to states. Previous studies predominantly focused on body image traits, that is, individuals’ consistent attitudes and evaluations of their bodies, while neglecting body image states, which represent immediate perceptions and evaluations of physical appearance at specific moments (Cash et al., 2002). By focusing on body image states, this study examines their associations with emotional eating, offering a new perspective for understanding the dynamic variability of body image and its behavioral correlates. This expansion helps capture the situational variability of body image in daily life, addressing the limitation that trait measurements struggle to reflect immediate experiences.

Second, this study integrates stress coping theory, the primary-secondary distinction in exercise addiction research, self-determination theory, and self-depletion theory to construct a comprehensive theoretical model. This model traces the pathway from cognitive evaluation to emotional experience, then to behavioral patterns, and ultimately to eating behaviors. Previous studies predominantly explained the relationship between body image and eating behaviors from a single theoretical perspective, rarely integrating multiple theories within a unified framework. Through this theoretical integration, the study identifies multiple pathways that are statistically supported in the association between body image state and emotional eating: via the affective pathway of perceived stress, the behavioral pathway of exercise addiction risk, and the sequential pathway linking both. This theoretical synthesis facilitates a more comprehensive understanding of the complex relationship between body image and eating behaviors.

Third, this study provides initial evidence consistent with a sequential pathway linking perceived stress and exercise addiction risk, responding to calls for greater attention to the interplay among mediating variables. Previous studies predominantly examined the independent effects of single mediators, with limited exploration of how multiple mediators may operate together. Grounded in the theoretical logic of stress coping theory, this research proposes and tests a model in which perceived stress is associated with exercise addiction risk, which in turn is associated with emotional eating. While the cross-sectional design cannot confirm the temporal order of these variables, the findings are consistent with the hypothesized sequence and suggest that emotional and behavioral pathways may be interconnected rather than merely parallel. Longitudinal research is needed to verify the proposed temporal ordering.

Fourth, this study contributes theoretically to the field of exercise addiction research. By examining the pathway to secondary exercise addiction risk, it identifies processes that are consistent with the view that body image concerns are associated with exercise addiction risk. This finding supports the core tenet of the primary-secondary distinction in exercise addiction research: when exercise serves external goals such as appearance enhancement, healthy exercise may be associated with addictive patterns (Weinstein and Szabo, 2023). Simultaneously, linking exercise addiction risk to emotional eating expands understanding of its correlates, suggesting that exercise addiction risk is not only associated with physical health but may also relate to eating behaviors through psychological resource depletion.

Fifth, this study extends the cultural context. Previous research on the relationship between exercise addiction risk and eating behaviors has predominantly been conducted in Western cultural settings. By using a sample of Chinese university students, this study examines the applicability of the theoretical model within an Eastern cultural context. This cross-cultural examination helps test the theory’s universality and provides a foundation for subsequent cross-cultural comparative research.

### 5.7 Practical implications

The findings of this study offer several implications for mental health promotion among college students. First, the identified serial pathway suggests that multi-component interventions addressing body image concerns, stress management, and exercise motivation simultaneously may be better positioned to reduce emotional eating than programs targeting any single factor alone. Second, the mediating role of perceived stress highlights the value of routine stress screening in university health services to identify students who may benefit from early intervention. Third, the association between exercise addiction risk and emotional eating suggests that health professionals should be attentive to the motivational quality of students’ exercise, distinguishing between flexible, intrinsically motivated physical activity and rigid, appearance-driven patterns. These implications are tentative given the cross-sectional design and require further evaluation in longitudinal and intervention research.

### 5.8 Research limitations and future directions

This study has several limitations that should be considered when interpreting the findings and that may guide future research.

First, limitations in research design. This study employed a cross-sectional design, collecting all variables at a single point in time, thus precluding the inference of causal relationships between variables. Although a sequential pathway was proposed based on theoretical reasoning—body image state associated with perceived stress associated with exercise addiction risk associated with emotional eating—cross-sectional data cannot confirm this temporal sequence. Consistent with this limitation, our tests of alternative models (parallel mediation and reversed serial mediation) also yielded significant indirect effects (Section 4.4). Thus, the data are consistent with multiple temporal orders, including the reverse order (body image state → exercise addiction risk → perceived stress → emotional eating). Therefore, the hypothesized serial pathway should be interpreted as a theoretically guided model that is statistically associated with the data, not as evidence of causal or temporal ordering. All reported indirect effects are statistical associations conditional on the assumed direction; they do not demonstrate that one variable precedes another in time. Future research using longitudinal designs with repeated measurements across multiple time points is necessary to examine directional relationships and to distinguish between competing mediation orders.

Second, limitations in measurement methods. All variables were collected via self-report questionnaires, which may introduce common method bias, social desirability, and recall bias. Although we implemented procedural remedies (anonymity, attention checks, reverse-scored items) and Harman’s single-factor test indicated no severe common method variance (28.8% < 40% threshold), such *post hoc* assessments do not fully eliminate concerns. Future research should integrate multi-method assessments, including ecological momentary assessment, physiological measures, and objective behavioral logs, to reduce bias and triangulate findings. To address these limitations, future studies should incorporate longitudinal designs, objective measures (e.g., exercise frequency logs, accelerometry), and multiple informants where possible.

Third, limitations in the sample. This study’s sample was restricted to college students, a group with relatively concentrated age ranges and lifestyles. Caution is warranted when generalizing findings to other populations. College students occupy a distinct developmental stage, potentially exhibiting differences in body image characteristics, stress sources, exercise habits, and dietary patterns compared to other age groups. Future research should expand samples to include adolescents, middle-aged adults, and older adults to examine the serial mediation model’s performance across developmental stages. Clinical samples, such as individuals with eating disorders or those seeking help for exercise addiction, could also be incorporated to assess the model’s clinical applicability.

Fourth, limitations in variable control. While we controlled for gender, grade level, and BMI, several other variables could potentially confound the observed associations. For example: baseline physical activity level and exercise frequency may correlate with both body image state (individuals who exercise more may have different body satisfaction) and exercise addiction risk (more exercise increases the opportunity for addiction); intrinsic vs. extrinsic exercise motivation is critical: individuals who exercise primarily for appearance or weight control may be at higher risk for secondary exercise addiction, whereas those exercising for enjoyment may not; social media use and appearance-related media exposure have been linked to body dissatisfaction and disordered eating; pre-existing mental health conditions (e.g., depression, anxiety, prior eating disorders) could influence all study variables; and self-esteem and emotion regulation strategies may independently affect both perceived stress and emotional eating. Because these variables were not measured, we cannot rule out the possibility that the observed indirect effects are partially or fully attributable to omitted confounders. Our estimates should therefore be interpreted as associations conditional on the covariates included, not as unbiased causal effects. Future research should measure these additional confounders and, if possible, employ longitudinal or experimental designs to better isolate the pathways examined here. Additionally, the cross-sectional design cannot rule out the possibility that greater exercise addiction risk is associated with higher exercise volume and increased physiological hunger rather than emotionally driven eating. Future studies incorporating objective exercise and dietary measures are needed to separate these pathways.

Fifth, limitations in model completeness. The serial mediation model examined here explains part of the mechanism linking body image state to emotional eating, yet some direct effects remain unexplained, suggesting potential additional mediating pathways. Future research could explore other potential mediating variables, such as self-esteem, self-objectification, social anxiety, and mindfulness levels, to enrich understanding of the processes through which body image relates to eating behaviors. Investigating moderators such as gender, social support, and emotion regulation strategies could also reveal how the model performs under different conditions.

Sixth, limitations regarding cultural factors. Conducted among Chinese university students, the findings may be influenced by specific cultural contexts. For instance, the internalization of thin-ideal appearance standards, which are amplified by social media, together with the compound pressures of academic and familial expectations, may heighten both body image concerns and perceived stress in this population. Additionally, sensitivity to social evaluation and the concept of “face” (mianzi) in collectivist contexts may increase the salience of appearance-related stressors. Future research should conduct cross-cultural comparisons to examine the applicability and variations of the proposed serial mediation model across different cultural backgrounds.

## 6 Conclusion

This study found a negative association between body image state and emotional eating among college students, with poorer body image states correlating with higher emotional eating tendencies. Perceived stress showed a mediating indirect effect in this relationship: body image state was associated with emotional eating through its association with elevated perceived stress levels, consistent with stress coping theories. Exercise addiction risk also showed a mediating indirect effect: body image state was associated with emotional eating through its association with secondary exercise addiction risk, supporting the primary-secondary distinction framework in exercise addiction research. The study further found evidence of a serial indirect effect of perceived stress and exercise addiction risk: body image state was associated with perceived stress, perceived stress was associated with exercise addiction risk, and exercise addiction risk was subsequently associated with emotional eating. This sequential pathway integrates stress coping theory, self-determination theory, and self-depletion theory, and is consistent with a psychological process from cognitive evaluation to emotional experience to behavioral patterns ultimately linked to eating behaviors.

The serial mediation model constructed and examined in this study offers new theoretical insights into the associations between body image states and emotional eating among college students. Findings suggest that body image states relate to eating behaviors not only through emotional pathways but also via the behavioral pathway of exercise addiction risk, with a sequential relationship between these two mechanisms. This finding broadens the research perspective on the relationship between body image and eating behaviors, shifting the focus from single mediating variables to sequential interactions among multiple variables, and provides an analytical framework for subsequent studies. The study also examines the applicability of the theoretical model within an Eastern cultural context, laying groundwork for future cross-cultural comparative research.

## Data Availability

The original contributions presented in the study are included in the article/Supplementary material, further inquiries can be directed to the corresponding author.

## Footnotes

1
https://www.wenjuan.com/s/UZBZJvYWmq/
